# Wall-Thickness-Dependent Microstructural Evolution and Mechanical Response of LPBF-Fabricated TA15 Titanium Alloy: The Role of Post-Solidification Cyclic Reheating

**DOI:** 10.3390/ma19112341

**Published:** 2026-06-01

**Authors:** Yunpeng Zhang, Zuo Li, Shilong Che, Xin Lin, Xufei Lu

**Affiliations:** 1 State Key Laboratory of Solidification Processing, Northwestern Polytechnical University, Xi’an 710072, China; zhypzhh@126.com (Y.Z.); lizuo@mail.nwpu.edu.cn (Z.L.); 2720175337@mail.nwpu.edu.cn (S.C.); xlin@nwpu.edu.cn (X.L.); 2 MIIT Key Laboratory of Metal High Performance Additive Manufacturing and Innovative Design, Northwestern Polytechnical University, Xi’an 710072, China

**Keywords:** laser powder bed fusion, TA15 titanium alloy, wall thickness, α′ martensite, mechanical properties

## Abstract

Wall thickness affects local heat accumulation during laser powder bed fusion (LPBF), but its role in governing the as-built martensitic morphology and tensile response of near-α TA15 alloy remains unclear. In this study, TA15 walls with thicknesses from 0.5 mm to 30 mm were fabricated under identical LPBF parameters. Optical microscopy, scanning electron microscopy, electron backscatter diffraction, tensile testing, fractography, and finite-element thermal simulation were used to correlate wall-thickness-dependent cyclic reheating with α′ lath evolution and mechanical behavior. Increasing wall thickness promoted α′ lath coarsening and the formation of colony-like lath structures with enlarged similarly oriented regions. The average α′ lath width increased from approximately 0.28 μm in the 0.5-T specimen to 1.55 μm in the 30-T specimen. The yield strength reached a maximum of 972.3 ± 5.29 MPa in the 1-T specimen, whereas elongation increased from 9.5 ± 0.6% to 17.8 ± 1.7% with increasing wall thickness. These results indicate a strong correlation between wall-thickness-dependent cyclic reheating, α′/α lath coarsening, lath-network evolution, and tensile-property variation in LPBF-fabricated TA15 alloy.

## 1. Introduction

TA15 (Ti-6.5Al-2Zr-1Mo-1V) is a near-α titanium alloy with a high aluminum equivalent and is widely used in aerospace load-bearing components because of its good specific strength, thermal stability, and creep resistance [1,2]. Conventional forging and thermomechanical processing can produce reliable TA15 components, but they are less efficient for thin-walled or variable-thickness structures with complex geometries. They also offer limited flexibility for local microstructure control [3]. Laser powder bed fusion (LPBF) provides a feasible route for manufacturing complex titanium alloy parts, while introducing rapid solidification and repeated thermal cycling during layer-wise melting [4,5,6].

For LPBF-fabricated TA15, cooling rates usually reach 10^5^–10^6^ K/s, leading to a non-equilibrium microstructure dominated by fine acicular α′ martensite within columnar prior-β grains [7,8,9,10]. This martensitic structure is one of the main reasons for the high strength of as-built TA15. The fine α′ laths and dense interfaces hinder dislocation motion, and tensile strengths above 1000 MPa have often been reported [7,11]. At the same time, the high density of interfaces, lattice defects, residual stresses, and possible pores restricts plastic deformation. As a result, the ductility of as-built LPBF TA15 is sensitive to α′ morphology, texture, residual stress state, and defect distribution [9,11,12,13,14,15].

Annealing and hot isostatic pressing are often used to improve the ductility and reliability of LPBF titanium alloys [5,16,17,18]. These treatments can reduce residual stress and defect sensitivity, and may promote the decomposition of α′ martensite into a more stable α + β microstructure [19,20,21,22,23]. However, post-treatments inevitably modify the as-built microstructure and may obscure the effect of thermal cycling generated during LPBF itself. For components used in the as-built or near-as-built condition, it is therefore important to distinguish whether the observed microstructural variation is associated with extensive α′ decomposition or with cyclic reheating experienced during layer-wise fabrication [24,25,26]. This issue is particularly relevant for thin-wall and variable-thickness structures, where local heat dissipation and heat accumulation can differ markedly across different geometric scales.

Besides processing parameters and post-treatment, component geometry also affects the thermal history in LPBF. Wall thickness is a typical example. A thin wall has a short heat dissipation path and a high surface-area-to-volume ratio, whereas a thick wall tends to accumulate heat and undergo longer cyclic reheating. Therefore, even with the same laser power, scanning speed, hatch spacing, and layer thickness, different wall thicknesses may produce different reheating temperatures, residence times, and cooling responses. These thermal differences can further affect α′ lath width, lath continuity, variant arrangement, and local deformation compatibility.

Previous studies on LPBF scale effects have shown that thin features and bulk regions may differ in melt-pool stability, surface morphology, cooling rate, and defect formation [27,28,29,30]. For titanium alloys, thinner regions generally favor finer martensitic structures, whereas thicker regions are more prone to heat accumulation and repeated reheating. Nevertheless, for near-α TA15 alloy, the mechanism by which wall thickness affects α′ martensitic morphology and tensile response remains insufficiently clarified. In particular, it is still unclear whether the wall-thickness effect is mainly related to extensive α′ martensite decomposition, post-solidification cyclic-reheating-induced lath coarsening, or the combined evolution of lath morphology, crystallographic arrangement, and deformation compatibility.

Most previous studies on LPBF-fabricated TA15 and related titanium alloys have focused on process optimization, general thermal accumulation, defect formation, residual stress, or post-treatment-induced microstructural modification. By contrast, the wall-thickness-dependent evolution of α′ lath width, lath-network continuity, crystallographic arrangement, and the associated strength–ductility balance under as-built cyclic reheating conditions has rarely been systematically examined over a wide thickness range. Therefore, a clearer understanding of the relationship among wall thickness, cyclic thermal exposure, α′ lath evolution, and tensile behavior is still needed for LPBF-fabricated near-α TA15 alloy.

In this study, TA15 wall structures with thicknesses ranging from 0.5 mm to 30 mm were fabricated by LPBF under identical processing conditions. OM, SEM, EBSD, KAM analysis, tensile testing, fractography, and finite-element (FE) thermal simulation were combined to examine the relationship among wall thickness, post-solidification cyclic reheating, α′ lath morphology, crystallographic features, and tensile response. The focus is placed on examining the relationship among wall-thickness-dependent cyclic reheating, α′/α lath morphology evolution, crystallographic features, and tensile response in LPBF-fabricated TA15 alloy. By correlating simulated reheating histories with lath morphology, deformation compatibility, and strength–ductility behavior, this work provides a more detailed understanding of the geometry-dependent microstructure–property relationship in as-built LPBF-fabricated near-α TA15 alloy.

## 2. Experimental Details

### 2.1. LPBF Fabrication Process

**Figure 1:**
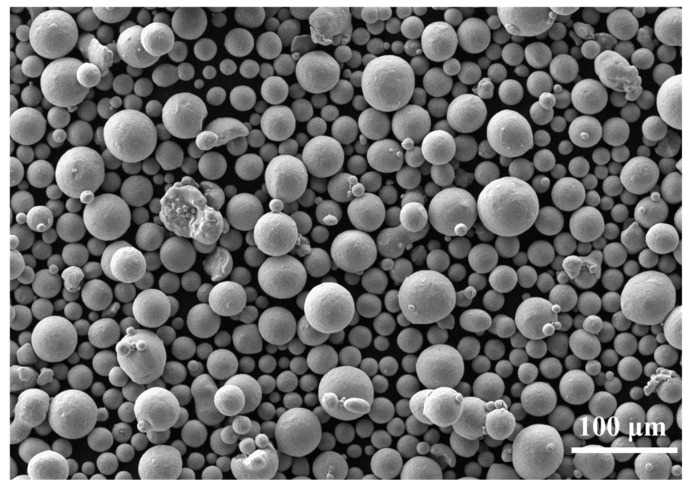
Surface morphology of the TA15 titanium alloy powder used for LPBF fabrication.

The TA15 titanium alloy powder used in this work was prepared by electrode induction melting gas atomization (EIGA), with a particle size distribution of 15–53 μm. Its nominal chemical composition is listed in Table 1, where Ti is the balance and Al, Zr, Mo, and V are the main alloying elements. The oxygen and nitrogen contents of the powder are 0.073 wt.% and 0.0092 wt.%, respectively. These interstitial elements are known to affect the strength and ductility of titanium alloys. Since all specimens in this work were fabricated using the same powder batch and identical LPBF processing conditions, the effects of oxygen and nitrogen contents are considered comparable among different wall-thickness conditions and are not expected to change the comparative trend associated with wall thickness. As shown in Figure 1, the powder particles are mostly spherical, with only a few satellite particles, which is favorable for powder spreading during LPBF. Before fabrication, the powder was dried in a vacuum oven at 120 °C for 2 h and then cooled to room temperature.

**Figure 2:**
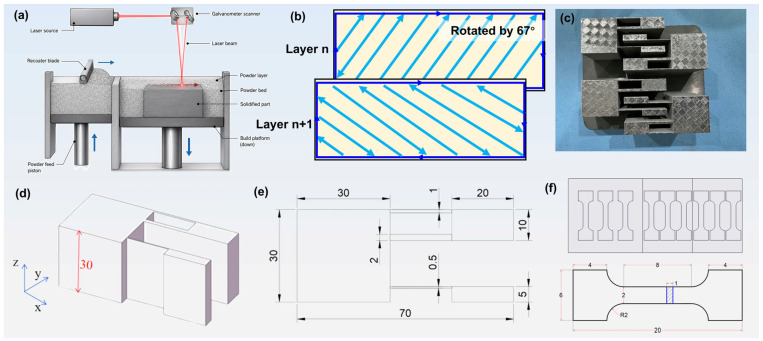
LPBF fabrication and specimen design: (**a**) schematic illustration of the LPBF process; (**b**) scanning strategy used in this work; (**c**) as-fabricated wall specimens with different thicknesses; (**d**,**e**) detailed dimensions of the specimens; and (**f**) dimensions of the tensile specimen.

Six groups of wall specimens with nominal thicknesses of 0.5, 1, 2, 5, 10, and 30 mm were fabricated using a BLT-S210 LPBF system (Xi’an Bright Laser Technologies, Xi’an, China) equipped with a 500 W ytterbium fiber laser, as schematically shown in Figure 2a. The specimens are denoted as 0.5-T, 1-T, 2-T, 5-T, 10-T, and 30-T according to their nominal designed wall thicknesses. All specimens were fabricated using identical LPBF parameters to isolate the effect of wall thickness on thermal history, microstructure, and tensile behavior. The laser power, scanning speed, hatch spacing, layer thickness, and laser spot diameter were 280 W, 1250 mm/s, 100 μm, 60 μm, and 100 μm, respectively. No substrate preheating was applied. A chessboard scanning strategy with a block size of 5 mm was used, and the scanning direction was rotated by 67° between adjacent layers to reduce directional stress accumulation, as shown in Figure 2b. The as-fabricated specimens are shown in Figure 2c, and their detailed dimensions are given in Figure 2d,e.

An annealed TC4 titanium alloy plate with dimensions of 108 × 127 × 20 mm^3^ was used as the substrate. Before LPBF processing, the substrate surface was mechanically ground to remove the oxide layer and then ultrasonically cleaned in acetone for 15 min.

### 2.2. Microstructure Characterization

The fabricated walls were separated from the substrate by wire electrical discharge machining (WEDM). Metallographic specimens were taken from the central region of each wall to reduce edge effects and to represent the internal microstructure. The specimens were mounted, ground sequentially with SiC papers from 400# to 3000#, and mechanically polished using diamond suspensions. For SEM observation, the polished specimens were etched for 1–2 s in a solution of 5 vol.% HF, 15 vol.% HNO_3_, and 80 vol.% H_2_O.

For EBSD analysis, the mechanically polished specimens were further electrolytically polished at −30 °C using an electrolyte of perchloric acid, n-butanol, and methanol with a volume ratio of 6:34:60. The polishing voltage was 25 V, and the polishing time was approximately 1 min. The low polishing temperature was selected to reduce the electrochemical reaction rate and suppress local heating, pitting, oxidation, and preferential dissolution during polishing, thereby improving EBSD indexing quality for the fine α′ lath structure. EBSD characterization was performed using a field-emission scanning electron microscope equipped with an EBSD detector (Oxford Instruments NanoAnalysis, Abingdon, UK). The EBSD scans were acquired with a step size of 0.08 μm over scan areas of approximately 37.1 μm × 29.1 μm. At least three representative regions were analyzed for each wall-thickness condition. Considering that the minimum average α′ lath width measured in this work is approximately 0.28 μm, the adopted step size provides sufficient spatial resolution to characterize the lath morphology and orientation features.

The EBSD data were used to analyze crystallographic orientation, phase distribution, and local misorientation. No confidence-index-based filtering or grain-dilation cleanup was applied during phase-fraction calculation, so as to avoid artificially redistributing weakly indexed pixels into the α or β phase. Unindexed pixels were retained in the analysis and are reported separately in Section 3.1. These unindexed regions mainly correspond to fine α′/α lath boundaries, areas with strong local orientation gradients, and regions with relatively low pattern quality. Kernel average misorientation (KAM) maps were calculated from the EBSD data to evaluate local lattice distortion.

### 2.3. Mechanical Testing

Tensile specimens were machined from the wall structures along the longitudinal direction in the XOZ plane, parallel to the build direction. The gauge section was positioned at the center of each wall to reduce the influence of surface roughness and edge-related geometric irregularity. As shown in Figure 2f, the specimens had a gauge length of 7 mm and an effective parallel length of approximately 8 mm.

All tensile specimens were machined into the same nominal geometry and tested using the same gripping configuration and tensile-testing procedure, regardless of wall thickness. This ensured consistent loading alignment and reliable comparison among different wall-thickness conditions. Room-temperature quasi-static tensile tests were performed according to ASTM E8 using an AGS-X universal testing machine (Shimadzu Corporation, Kyoto, Japan) equipped with a non-contact video extensometer. The nominal strain rate was 1 × 10^−3^ s^−1^.

Before testing, each specimen was carefully mounted to minimize bending or misalignment during loading. The specimen dimensions in the gauge section were measured before each test and used by the tensile-testing system to calculate the engineering stress. The elongation was measured within the gauge section using the non-contact video extensometer (Shimadzu Corporation, Kyoto, Japan), thereby reducing possible errors associated with fixture compliance or grip displacement. The 0.2% offset yield strength, ultimate tensile strength, and elongation were obtained from the engineering stress–strain curves. At least three independent tensile specimens were tested for each wall-thickness condition.

### 2.4. Thermal Simulation

A three-dimensional FE thermal model was established to calculate the transient temperature evolution during LPBF fabrication of TA15 walls with different thicknesses. The model geometry, meshing, and post-processing were performed using the GiD pre-/post-processing platform, while the thermal calculation was implemented using the in-house FE code COMET, following the numerical framework reported in our previous studies [32,33]. The simulation was designed to compare the relative heat accumulation and post-solidification cyclic reheating behavior among wall specimens with different thicknesses. It was not intended to resolve melt-pool-scale fluid flow, keyhole behavior, latent heat release, or detailed solidification dynamics. Therefore, the calculated temperature histories should be interpreted as qualitative or semi-quantitative indicators of relative thermal exposure rather than precise absolute temperature predictions. The calculated thermal histories were subsequently correlated with the measured α′ lath morphology and tensile response.

The transient heat-transfer problem was solved during layer-wise material deposition. Heat generated by plastic deformation was neglected because it is much smaller than the laser energy input during LPBF. The latent heat associated with melting and solidification was not explicitly included. This simplification may affect the calculated absolute peak temperatures during the initial melting/solidification stage. However, because the present analysis focuses on the relative differences in post-solidification reheating behavior among different wall thicknesses, rather than on the detailed melt-pool-scale solidification process, the model is considered suitable for comparative thermal-history analysis.

**Figure 3:**
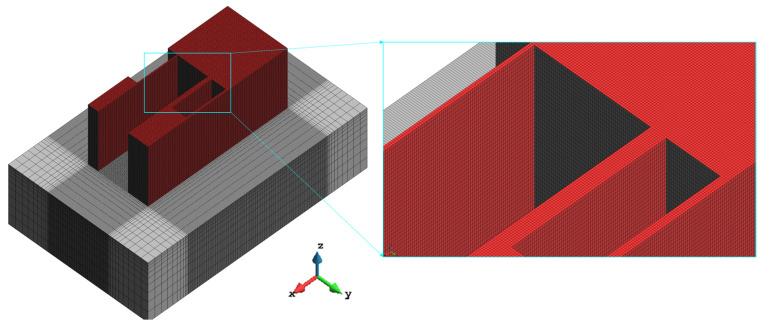
FE model consisting of the deposited wall structures and the substrate.

The FE model consisted of the deposited wall structures and the substrate, as shown in Figure 3. Six wall thicknesses, namely 0.5, 1, 2, 5, 10, and 30 mm, were considered. The deposited region was discretized using hexahedral elements with a nominal size of 0.25 × 0.25 × 0.5 mm^3^, which was selected based on a mesh-sensitivity check. The substrate mesh was gradually coarsened away from the deposited region to improve computational efficiency while maintaining sufficient resolution near the wall–substrate interface. The complete model contained approximately 1,639,200 hexahedral elements and 1,727,211 nodes. Element activation was adopted to reproduce the layer-wise material addition during LPBF [4].

The thermal boundary conditions were selected according to the dominant heat-transfer routes in the LPBF process. During fabrication, heat is mainly conducted from the newly deposited material to the previously solidified layers and then to the substrate, while heat loss from the exposed wall surfaces occurs through convection and radiation to the surrounding atmosphere. Accordingly, the temperature field was governed by laser energy input, heat conduction within the deposited material and substrate, and surface heat loss through convection and radiation. The laser absorption efficiency was set to 40% [34]. Heat extraction through the substrate was treated as the dominant heat-loss path, and an equivalent heat-transfer coefficient of 1000 W/(m^2^·°C) was applied to the bottom surface of the substrate to represent heat dissipation into the build platform. Heat loss from the exposed wall surfaces was modeled using combined convection and radiation, with a convection coefficient of 12.7 W/(m^2^·°C), an ambient temperature of 24 °C, and a surface emissivity of 0.35. These boundary conditions were selected to capture the relative differences in heat accumulation and cyclic reheating among different wall thicknesses, rather than to predict the exact melt-pool-scale temperature field. The temperature-dependent thermophysical properties of TA15 and Ti6Al4V used in the simulation are listed in Table 2 and Table 3, respectively [30,35].

For each wall thickness, one representative monitoring point was selected at the geometric center of the wall, corresponding to the mid-height position along the build direction and the center of the horizontal cross-section. This position was chosen to reduce the influence of free surfaces and edge-related thermal fluctuations, thereby enabling a consistent comparison of internal thermal histories among different wall-thickness conditions. The thermal histories extracted from these points were used to compare the reheating temperature, cooling response, and cumulative elevated-temperature exposure experienced by previously solidified regions. These quantities were then used to interpret the wall-thickness-dependent α′ lath coarsening and the associated change in tensile behavior.

It should be emphasized that the present FE thermal model is simplified and mainly intended for comparative analysis of wall-thickness-dependent heat accumulation. Direct experimental validation of the transient temperature histories was not performed in this work. In addition, latent heat, melt-pool fluid flow, keyhole behavior, and detailed solidification dynamics were not explicitly considered. Therefore, the calculated peak temperatures should not be regarded as highly precise absolute values. Instead, emphasis is placed on the relative differences in post-solidification cyclic reheating behavior among specimens with different wall thicknesses. This interpretation is consistent with the purpose of the simulation, namely to support the discussion of geometry-dependent thermal exposure and its correlation with α′/α lath morphology evolution.

## 3. Results and Discussion

### 3.1. Microstructural Evolution at Different Wall Thicknesses

**Figure 4:**
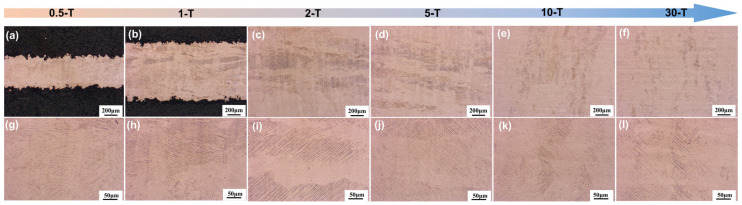
Optical micrographs of LPBF-fabricated TA15 alloy walls with different thicknesses. (**a**–**f**) Cross-sectional morphologies of 0.5-T, 1-T, 2-T, 5-T, 10-T, and 30-T specimens, respectively; (**g**–**l**) corresponding high-magnification microstructures.

Before discussing the microstructural evolution, the metallurgical quality of the LPBF-fabricated TA15 walls was first evaluated using polished cross-sectional OM images, as shown in Figure 4a–f. Overall, all specimens exhibit relatively dense metallurgical structures without obvious lack-of-fusion defects or large keyhole pores. Only a limited number of small pores with sizes of approximately 3–5 μm can be observed in the 0.5-T and 1-T specimens, whereas such pores become much less apparent when the wall thickness increases to 2 mm and above. These observations suggest that large metallurgical defects are unlikely to dominate the overall tensile-property variation under the present processing conditions. Nevertheless, the ultra-thin-wall specimens remain more sensitive to local thermal instability, contour fluctuation, and surface/edge-related imperfections. The polished cross-sectional OM images were also examined to evaluate the metallurgical quality of the LPBF-fabricated walls. No obvious large lack-of-fusion defects or keyhole pores were observed in the investigated sections. However, the ultra-thin-wall specimens exhibit greater local contour fluctuation and dimensional instability compared with the thicker-wall specimens, which may increase their sensitivity to surface/edge-related imperfections during tensile loading.

Figure 4g–l shows the corresponding high-magnification microstructures. All specimens exhibit a layered morphology along the build direction, which is consistent with the layer-wise nature of LPBF [36,37,38]. However, both the wall contour and the internal microstructure change markedly with wall thickness. For the 0.5-T specimen, the wall boundary is relatively rough and irregular, indicating a pronounced thin-wall effect and less stable melt-pool behavior. Such contour irregularity may introduce local surface/edge-related stress concentration during tensile loading. When the wall thickness increases to 1 mm, the wall geometry becomes more regular, and the layer bands are more continuous. For the 2-T to 30-T specimens, the cross-sectional morphology becomes more stable, and continuous melt-pool traces can be more clearly identified along the build direction. These observations indicate that increasing wall thickness improves geometric stability and metallurgical continuity, while also changing the local heat dissipation condition and promoting greater heat accumulation during fabrication.

At higher magnification, all specimens are dominated by acicular or lath-like α′ martensite formed under rapid solidification. The 0.5-T specimen contains very fine α′ laths, but the lath network appears fragmented and discontinuous. In the 1-T specimen, the fine α′ laths are more continuous and uniformly interwoven. With increasing wall thickness to 2-T and 5-T, the laths become longer and thicker, and locally parallel lath groups begin to appear. In the 10-T and 30-T specimens, the α′ laths are markedly coarsened and arranged into bundles with similar orientations. The density of lath boundaries is reduced, and the microstructure changes from a fine interwoven morphology to a more colony-like configuration.

The OM observations therefore indicate a wall-thickness-dependent change in both metallurgical quality and α′ martensitic morphology. Increasing wall thickness promotes α′ lath coarsening and the development of similarly oriented lath bundles, implying that the local thermal history differs among walls fabricated using the same nominal LPBF parameters. Meanwhile, the relatively rough contour, occasional small pores, and fragmented lath network observed in the 0.5-T specimen suggest that its mechanical response may not be governed by α′ lath refinement alone. Thin-wall instability and surface/edge-related imperfections may also contribute to local stress concentration and reduced strengthening efficiency. This point is further discussed in relation to tensile behavior in Section 3.5.

**Figure 5:**
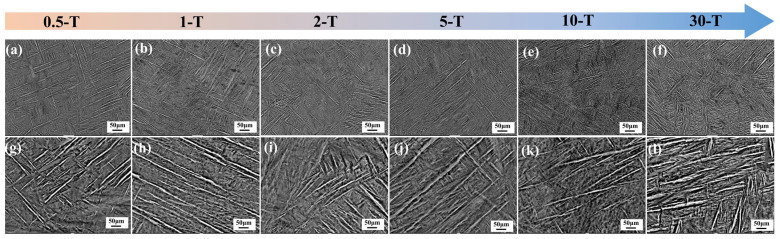
SEM images of LPBF-fabricated TA15 alloy walls with different thicknesses. (**a**–**f**) Low-magnification images of 0.5-T, 1-T, 2-T, 5-T, 10-T, and 30-T specimens, respectively; (**g**–**l**) corresponding high-magnification images.

SEM observations were further used to examine the α′ lath morphology, as shown in Figure 5. The SEM images confirm that all specimens are mainly composed of acicular α′ martensite, while the lath width, continuity, and arrangement vary with wall thickness.

In the 0.5-T specimen (Figure 5a,g), the α′ laths are very fine, with an average width of approximately 0.28 ± 0.07 μm, but the lath network is relatively fragmented. In the 1-T specimen (Figure 5b,h), the laths coarsen slightly to 0.42 ± 0.10 μm and form a dense, continuous, and interwoven structure. This morphology gives the 1-T specimen a high lath-boundary density while maintaining good structural continuity.

When the wall thickness increases to 2 mm (Figure 5c,i), the average lath width increases to 0.60 ± 0.15 μm, and locally parallel lath arrangements begin to develop. In the 5-T specimen (Figure 5d,j), the laths further coarsen to 0.85 ± 0.20 μm, together with the formation of more distinct lath bundles or colonies. This indicates that the microstructure begins to shift from a highly interwoven lath network to a coarser, more organized morphology.

For the 10-T specimen (Figure 5e,k), the α′ laths coarsen substantially to 1.20 ± 0.25 μm and are mainly arranged as large parallel bundles. In the 30-T specimen (Figure 5f,l), the average lath width further increases to 1.55 ± 0.35 μm. Compared with the change from 0.5-T to 10-T, the increase from 10-T to 30-T is less pronounced, suggesting that lath coarsening tends to slow down at large wall thickness. The 30-T specimen shows the largest similarly oriented regions and the lowest apparent lath-boundary density.

The SEM measurements show a monotonic increase in α′ lath width from 0.28 μm to 1.55 μm as the wall thickness increases from 0.5 mm to 30 mm. This confirms that wall thickness modifies the thermal history sufficiently to change the α′ lath morphology, even when the nominal LPBF parameters are unchanged.

**Figure 6:**
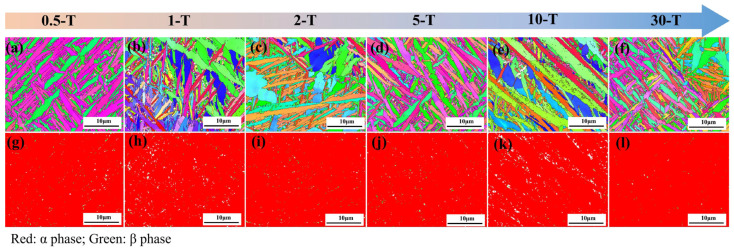
EBSD characterization of LPBF-fabricated TA15 alloy walls with different thicknesses. (**a**–**f**) IPF maps and (**g**–**l**) corresponding phase maps for 0.5-T, 1-T, 2-T, 5-T, 10-T, and 30-T specimens.

EBSD analysis was carried out to examine the crystallographic features and phase constitution, as shown in Figure 6. The inverse pole figure (IPF) maps (Figure 6a–f) show that all specimens contain fine lath-like α/α′ structures with multiple crystallographic orientations. Since α and α′ have the same hcp crystal structure, EBSD phase indexing alone cannot unambiguously distinguish martensitic α′ from equilibrium α. The EBSD phase results are therefore interpreted together with the SEM morphology and the simulated thermal histories.

The IPF maps reveal a thickness-dependent change in lath orientation arrangement. The 0.5-T specimen exhibits fragmented and randomly oriented laths, showing a strong variant-interweaving feature. The 1-T specimen has a more continuous lath network while retaining a high density of orientation variants. As the wall thickness increases to 2-T and 5-T, locally parallel lath groups become more evident, indicating the onset of lath-bundle formation and reduced variant randomness.

For the 10-T and 30-T specimens, larger regions with similar crystallographic orientations are observed, corresponding to well-developed lath bundles and enlarged colony structures. This crystallographic feature agrees with the SEM observations and confirms the transition from a highly interwoven microstructure to a more orientation-correlated morphology with increasing wall thickness.

The phase maps (Figure 6g–l) show that the indexed α phase dominates all specimens, while only a small amount of β phase is detected sporadically. As summarized in Table 4, the EBSD indexing rate remains higher than 96% for all wall-thickness conditions, ranging from 96.8% to 99.9%. The indexed α phase fraction remains above 96%, whereas the β phase fraction stays at a low level of approximately 0.2–0.5% across the investigated wall-thickness range. The α and β phase fractions do not strictly sum to 100% because a small fraction of pixels could not be reliably indexed during EBSD analysis.

No confidence-index-based filtering or grain-dilation cleanup was applied during phase-fraction calculation. Instead, the unindexed pixels were retained and reported separately in Table 4 to reflect the actual indexing condition of the EBSD data. These unindexed pixels are mainly associated with fine α′/α lath boundaries, local orientation gradients, and low-pattern-quality regions, which are commonly observed in LPBF-fabricated martensitic titanium alloys. This treatment avoids artificially redistributing weakly indexed pixels into the α or β phase and therefore prevents overestimation of the indexed phase fractions.

No systematic increase in β phase fraction is observed with increasing wall thickness. Combined with the SEM observations and simulated thermal histories, this result indicates that the wall-thickness-dependent microstructural evolution is strongly associated with changes in α′/α lath morphology, lath arrangement, and cyclic reheating history. Therefore, within the resolution of the present characterization methods, the observed wall-thickness effect is mainly reflected by the coarsening, alignment, and colony formation of the α′/α lath structure under different cyclic thermal histories. The corresponding variation in mechanical properties is therefore more closely related to lath morphology and deformation compatibility than to a major change in phase constitution.

### 3.2. Wall-Thickness-Dependent Tensile Properties

**Figure 7:**
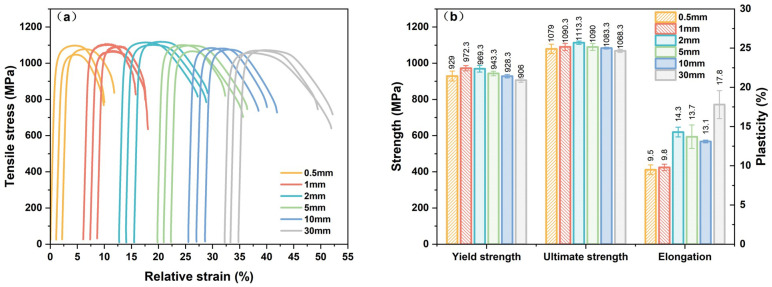
Tensile properties of LPBF-fabricated TA15 alloy walls with different thicknesses: (**a**) engineering stress–strain curves corresponding to all tensile tests conducted for each wall-thickness condition; and (**b**) yield strength, ultimate tensile strength, and elongation. The error bars represent the standard deviations obtained from at least three repeated tensile tests.

Figure 7 shows the tensile properties of LPBF-fabricated TA15 alloy walls with different thicknesses. The engineering stress–strain curves in Figure 7a correspond to all tensile tests conducted for each wall-thickness condition, rather than selected representative curves. The corresponding yield strength, ultimate tensile strength, and elongation are summarized in Figure 7b, where the error bars represent the standard deviations obtained from repeated tests. The average tensile properties and corresponding standard deviations are further listed in Table 5. All specimens exhibit an elastic–plastic response followed by strain hardening before fracture, but the strength–ductility balance varies clearly with wall thickness.

As shown in Table 5, the yield strength first increases from 929 ± 27.2 MPa for the 0.5-T specimen to 972.3 ± 5.29 MPa for the 1-T specimen, and then gradually decreases to 906 ± 11.3 MPa for the 30-T specimen. The ultimate tensile strength remains at a relatively high level for the thin- and medium-thickness walls, with values of 1079 ± 25.6 MPa, 1090.3 ± 5.5 MPa, and 1113.3 ± 9.0 MPa for the 0.5-T, 1-T, and 2-T specimens, respectively, followed by a gradual decrease to 1068.3 ± 8.1 MPa for the 30-T specimen. Thus, the 1-T specimen exhibits the highest average yield strength, whereas the highest average ultimate tensile strength is obtained in the 2-T specimen. However, the UTS differences among the 1-T, 2-T, and 5-T specimens are relatively small compared with the overall variation in elongation. Therefore, the following discussion focuses mainly on the overall strength–ductility evolution with wall thickness, rather than treating every minor difference between neighboring conditions as statistically distinct.

The elongation shows a different trend from strength. The 0.5-T specimen has the lowest elongation of 9.5 ± 0.6%, and the 1-T specimen shows a similar value of 9.8 ± 1.4%. When the wall thickness increases to 2 mm, the elongation increases to 14.3 ± 0.6%. The 5-T and 10-T specimens remain at comparable levels of 13.7 ± 1.5% and 13.1 ± 0.2%, respectively, whereas the 30-T specimen reaches the highest elongation of 17.8 ± 1.7%. These results indicate that ductility is generally improved with increasing wall thickness, although minor fluctuations exist among the medium-thickness specimens.

The above results demonstrate that wall thickness changes the strength–ductility balance of LPBF-fabricated TA15 alloy. The 1-T specimen combines fine α′ laths with a relatively continuous lath network, which is favorable for achieving high yield strength. Although the 2-T specimen shows the highest average ultimate tensile strength, its difference from the 1-T specimen is limited. By contrast, thicker specimens generally show lower strength but better ductility, which is consistent with their coarser α′ laths and more developed lath bundles. The 0.5-T specimen, although having the finest α′ laths, does not exhibit the highest strength. This result indicates that α′ lath refinement alone is insufficient to determine the tensile strength of ultra-thin-wall specimens. In addition to the fragmented lath network, the larger dimensional fluctuation, reduced gauge cross-sectional stability, local contour irregularity, and possible surface/edge-related imperfections in the 0.5-T specimen may weaken the effective strengthening contribution and promote local stress concentration during tensile loading.

### 3.3. Fracture Behavior at Different Wall Thicknesses

**Figure 8:**
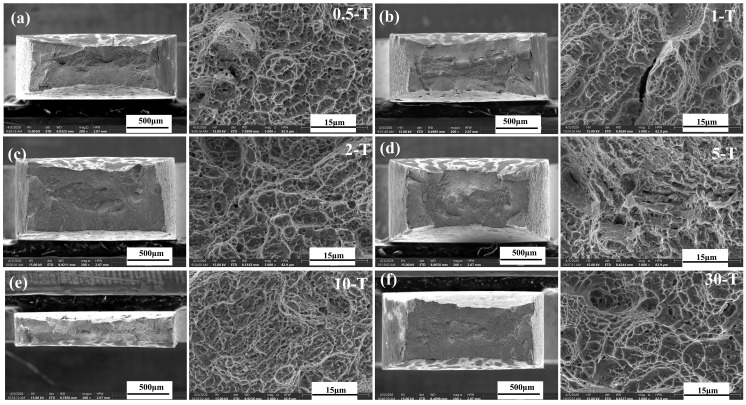
Fracture surfaces of LPBF-fabricated TA15 alloy walls with different thicknesses after tensile testing: (**a**) 0.5-T; (**b**) 1-T; (**c**) 2-T; (**d**) 5-T; (**e**) 10-T; and (**f**) 30-T.

The fracture morphologies of LPBF-fabricated TA15 alloy walls after tensile testing are shown in Figure 8. At low magnification (Figure 8a–f), all specimens show rough fracture surfaces without obvious cleavage facets or macroscopic brittle-fracture features, indicating an overall ductile fracture mode. No large lack-of-fusion defects, keyhole pores, or macroscopic brittle-fracture regions are observed on the examined fracture surfaces. High-magnification observations further reveal dimpled fracture surfaces, suggesting that fracture occurs mainly through microvoid nucleation, growth, and coalescence. Nevertheless, the dimple morphology and possible damage-initiation features vary with wall thickness.

For the 0.5-T specimen (Figure 8a), the fracture surface contains fine but non-uniform dimples, with several shallow-dimple regions. This indicates limited plastic deformation before fracture. Although no large lack-of-fusion defects or keyhole pores are observed, the ultra-thin 0.5-T specimen is more sensitive to thin-wall instability, surface/edge irregularities, and occasional small pores. These features may act as local stress-concentration sites and facilitate early microvoid nucleation during tensile loading. This provides a possible explanation for why the 0.5-T specimen does not exhibit the highest strength despite having the finest α′ laths. The 1-T specimen (Figure 8b) also shows fine and densely distributed dimples, but the dimples remain relatively shallow, which agrees with its high strength and limited elongation. For both thin-wall specimens, the fracture behavior is therefore considered to be affected by the combined effects of fine α′ lath morphology, limited plastic accommodation, and possible surface/edge-related damage initiation.

When the wall thickness increases to 2-T and 5-T (Figure 8c,d), the dimples become larger and deeper, and their distribution becomes more uniform, reflecting improved plastic-deformation capacity. This indicates that the thicker walls can accommodate plastic strain more effectively before final fracture. For the 10-T specimen (Figure 8e), the fracture surface contains more developed dimples with relatively homogeneous distribution. The 30-T specimen (Figure 8f) shows the most evident ductile-fracture morphology, with large, deep, and relatively equiaxed dimples over the fracture surface. This is consistent with the highest elongation measured for this specimen. The coarsened α′ laths and enlarged similarly oriented regions in thicker specimens provide longer and more continuous slip paths, while the reduced lath-boundary density decreases local strain incompatibility. These microstructural features promote more uniform plastic deformation and delay final fracture.

Although all specimens fail mainly by ductile fracture, the dimple morphology changes from fine and shallow in thin walls to larger and deeper in thick walls. This trend is consistent with the tensile results and indicates that increasing wall thickness improves the ability of the microstructure to accommodate plastic deformation. It should be noted that no obvious large lack-of-fusion defects, large keyhole pores, or cleavage-like brittle-fracture regions were identified on the examined fracture surfaces. However, the present SEM fractography does not allow a unique fracture-initiation site to be unambiguously determined. Therefore, the lower ductility of the 0.5-T and 1-T specimens should not be attributed to a single fracture-origin mechanism. Instead, it may be associated with the combined effects of dense α′/α lath-boundary networks, limited deformation compatibility, dimensional instability, and increased sensitivity to possible surface/edge-related imperfections in ultra-thin-wall specimens. Further high-resolution fracture-origin analysis and defect tracking, such as side-surface observation near the fracture region or X-ray CT characterization, are needed to identify the exact fracture-initiation sites. For the 0.5-T and 1-T specimens, local surface/edge irregularities and occasional small pores may contribute to earlier damage initiation, although no dominant large defect is identified on the examined fracture surfaces. In contrast, the thicker specimens exhibit more developed dimple morphology, suggesting more stable plastic deformation before final failure.

It should be noted that systematic crack-initiation tracing and quantitative dimple-size statistics were not performed in the present work. Therefore, the fracture-surface observations are used mainly to support the qualitative interpretation of tensile deformation behavior, rather than to provide a quantitative fracture-initiation criterion. Overall, the fracture morphologies suggest that the tensile deformation behavior of LPBF-fabricated TA15 alloy is closely associated with the deformation accommodation capability of the α′ lath structure. The transition from fine and shallow dimples to larger and deeper dimples with increasing wall thickness reflects the gradual improvement in plastic-deformation compatibility during tensile loading.

### 3.4. Thermal Origin of α′ Lath Coarsening

**Figure 9:**
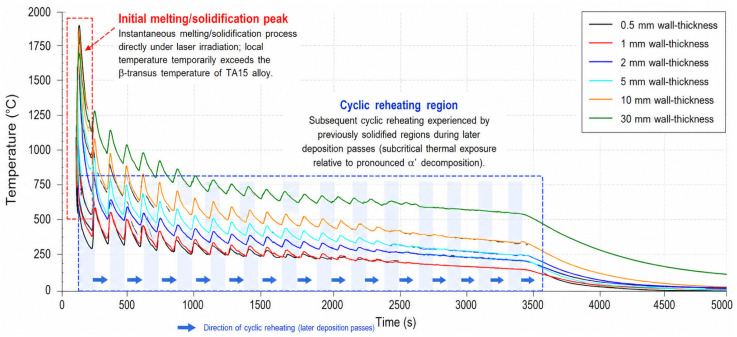
Simulated thermal-history curves extracted from the geometric center of LPBF-fabricated TA15 alloy walls with different thicknesses. The monitoring point is located at the mid-height position along the build direction and at the center of the horizontal cross-section of each wall. The high initial peak corresponds to the instantaneous melting/solidification process under direct laser irradiation, whereas the marked region indicates the subsequent post-solidification reheating cycles experienced by previously solidified material during later deposition passes.

The coarsening of α′ martensitic laths is closely related to the cyclic thermal exposure imposed during LPBF. In this work, a post-solidification reheating cycle refers to one reheating-and-cooling event experienced by previously solidified material during subsequent layer deposition. This definition is used to distinguish the subsequent reheating oscillations from the initial high-temperature melting/solidification peak directly induced by laser irradiation. The initial peak mainly represents transient melting and solidification, whereas the later oscillatory temperature response reflects repeated reheating of already solidified material and is used here only for comparative analysis of thermal accumulation among different wall thicknesses. Figure 9 shows the simulated thermal histories extracted from the geometric center of walls with different thicknesses. Since the present thermal model was established for comparative heat-accumulation analysis, the calculated temperatures are interpreted as qualitative to semi-quantitative indicators of relative reheating behavior, rather than as exact absolute temperature values.

The high initial temperature peak in each thermal-history curve corresponds to the instantaneous melting/solidification process directly induced by laser irradiation. During this stage, the local temperature can temporarily exceed the β-transus temperature of TA15 alloy. By contrast, the temperature range discussed below refers to the subsequent reheating cycles experienced by previously solidified material during later deposition passes. In this work, a thermal cycle denotes one reheating-and-cooling event imposed on previously solidified material during subsequent layer deposition. These post-solidification reheating cycles provide the thermal basis for geometry-dependent heat accumulation and α′ lath evolution during LPBF.

For the 0.5-T specimen, the simulated reheating temperature during cyclic reheating is approximately 150–250 °C, and the elevated-temperature residence time is very short, ranging from milliseconds to about 1 s. Under this condition, heat accumulation is limited, and thermally activated recovery or lath-boundary migration is weak. As a result, very fine α′ laths are retained. However, the lath network is relatively fragmented, which is likely associated with rapid heat dissipation, pronounced thin-wall effects, and unstable local thermal conditions.

For the 1-T specimen, the simulated reheating temperature increases to approximately 200–300 °C, with a residence time of about 1–3 s. This slightly increased thermal exposure remains insufficient to cause pronounced phase transformation, but it may promote limited stress relaxation and improve lath-network continuity. Therefore, the 1-T specimen retains a fine α′ martensitic structure while developing a more continuous and uniformly interwoven lath morphology.

When the wall thickness increases to 2 mm, the estimated reheating temperature reaches approximately 250–350 °C, and the residence time extends to about 2–5 s. The increased thermal exposure provides greater opportunity for local recovery, interface adjustment, and limited lath rearrangement. This is consistent with the appearance of locally parallel lath groups in the 2-T specimen, indicating the early transition from a highly interwoven lath structure to a more organized lath arrangement.

For the 5-T specimen, the estimated reheating temperature further increases to approximately 300–430 °C, and the residence time reaches about 5–15 s. At this stage, the effect of heat accumulation becomes more apparent. Repeated reheating promotes local recovery, interface migration, and lath thickening, leading to more evident α′ lath coarsening and the formation of lath bundles. Consequently, the microstructure begins to shift from a fine interwoven lath network to a coarser and more organized morphology.

For the 10-T specimen, the estimated reheating temperature is approximately 350–500 °C, with a residence time of about 10–30 s. The prolonged thermal exposure further promotes α′ lath coarsening and lath-bundle growth. Correspondingly, larger similarly oriented regions are formed, and the lath-boundary density decreases. This indicates that increased wall thickness enhances heat accumulation and facilitates the development of orientation-correlated lath structures.

For the 30-T specimen, the estimated reheating temperature increases to approximately 400–550 °C, and the cumulative elevated-temperature exposure extends to several tens of seconds or longer owing to repeated thermal cycling. Although this temperature range remains below that typically required for extensive α′ martensite decomposition, the prolonged post-solidification exposure can still provide sufficient thermal activation for lath thickening, interface migration, recovery, and variant coalescence. This explains the coarsest α′ lath morphology and the most developed colony-like structure observed in the 30-T specimen.

Overall, increasing wall thickness changes the local thermal condition from rapid heat dissipation to progressive heat accumulation. This change increases both the simulated reheating temperature and the elevated-temperature residence time experienced by previously solidified regions. Consequently, α′ lath coarsening, lath rearrangement, and variant coalescence are progressively promoted, leading to the transition from a fine interwoven lath structure in thin walls to a coarse colony-dominated morphology in thick walls.

It should be emphasized that the simulated post-solidification reheating cycles are estimated to remain below the typical temperature range generally associated with pronounced α′ → α + β transformation in titanium alloys [39]. Meanwhile, the EBSD results show that the indexed α phase remains dominant and that the β phase fraction remains low without a systematic increase with wall thickness. However, because EBSD cannot unambiguously distinguish α from α′ and may not fully resolve nanoscale or boundary-localized β phases, these results should not be interpreted as definitive evidence of the local α′ decomposition. Instead, the present observations mainly suggest that the wall-thickness-dependent microstructural variation is closely correlated with changes in α′/α lath morphology, lath arrangement, interface migration, and colony evolution during post-solidification cyclic reheating.

### 3.5. Strength–Ductility Relationship and Underlying Mechanisms

The variation in strength and ductility with wall thickness can be linked to the evolution of α′ lath width, boundary density, lath-network continuity, and lath arrangement. A quantitative comparison between average α′ lath width and tensile strength shows that the α′ lath width increases from approximately 0.28 μm in the 0.5-T specimen to 1.55 μm in the 30-T specimen. Correspondingly, the yield strength first increases from 929 MPa for the 0.5-T specimen to 972.3 MPa for the 1-T specimen, and then gradually decreases to 906 MPa for the 30-T specimen. This trend indicates that finer α′ laths and higher lath-boundary density generally enhance dislocation blocking, which is consistent with a Hall–Petch-type strengthening concept. However, the strength does not vary monotonically with the average α′ lath width, indicating that α′ lath width alone cannot fully describe the tensile response of LPBF-fabricated TA15 alloy. Other factors, including lath-network continuity, thin-wall stability, defect sensitivity, and deformation compatibility, must also be considered.

The strengthening effect is closely related to both the density and effectiveness of α′ lath boundaries. In the 1-T specimen, the α′ laths are fine and continuous, providing a high density of effective barriers to dislocation motion. This accounts for its highest yield strength among the investigated conditions. Although the 0.5-T specimen has the finest α′ laths, its strength is lower than that of the 1-T specimen. This deviation indicates that the strengthening behavior cannot be described by a simple Hall–Petch-type relationship based only on the average α′ lath width.

For the ultra-thin 0.5-T specimen, the α′ lath network is relatively fragmented and discontinuous, which may reduce the effectiveness of lath-boundary strengthening. In addition, the 0.5-T wall exhibits a rougher contour and a larger dimensional deviation, making it more susceptible to local thermal instability and surface/edge-related stress concentration during tensile loading. Occasional small pores may further contribute to local damage initiation, although no large lack-of-fusion defects or keyhole pores are observed. Therefore, porosity is not considered to be the primary factor governing the overall strength–ductility trend, but thin-wall-related imperfections may partly explain why the 0.5-T specimen shows lower strength than the 1-T specimen despite having finer α′ laths. It should also be noted that X-ray CT and systematic surface roughness measurements were not performed in the present work. Therefore, the individual contributions of internal defects, surface quality, dimensional stability, and microstructural morphology to the tensile behavior of ultra-thin-wall specimens require further quantitative investigation in future studies.

When the wall thickness increases from 2-T to 30-T, the α′ laths become progressively coarser and more frequently arranged into bundles or colony-like regions. The resulting decrease in lath-boundary density reduces the resistance to dislocation motion, leading to the gradual decline in yield strength. It should be noted that the 2-T specimen shows the highest average ultimate tensile strength, while the UTS differences among the 1-T, 2-T, and 5-T specimens are relatively small. Therefore, the strength discussion focuses mainly on the overall trend of yield-strength reduction and ductility improvement with increasing wall thickness, rather than treating every minor difference in UTS as statistically distinct. Taken together, the tensile strengthening behavior of LPBF-fabricated TA15 alloy is jointly governed by α′ lath refinement, lath-network continuity, defect sensitivity, and deformation compatibility.

The ductility follows a different trend. In thin walls, the dense lath-boundary network shortens the effective slip length and restricts dislocation glide. This promotes local strain incompatibility and early microvoid formation, thereby limiting elongation. In thicker walls, the coarsened α′ laths and enlarged similarly oriented regions provide longer and more continuous slip paths. The reduced lath-boundary density also decreases local strain incompatibility across adjacent laths. As a result, plastic deformation can be accommodated more uniformly, which is consistent with the increased elongation and the fracture-surface transition from relatively shallow dimples in thin walls to larger and deeper dimples in thick walls.

The EBSD phase results further show that the β phase fraction remains very low and does not increase systematically with wall thickness. Therefore, the change in tensile properties is unlikely to be dominated by extensive phase-transformation-induced strengthening or softening. Instead, the tensile response is more closely associated with the evolution of α′/α lath morphology, lath-boundary density, lath-network continuity, and deformation compatibility under different post-solidification cyclic thermal histories.

Therefore, the dominant factor is considered to be the thermal-history-dependent evolution of α′ lath morphology and lath-network architecture. Fine and continuous α′ laths provide effective lath-boundary strengthening, whereas coarser laths and colony-like arrangements improve deformation compatibility. The KAM/GND results thus help exclude a systematic difference in initial local lattice distortion as the main cause and further support the interpretation that α′ lath coarsening, lath arrangement evolution, and deformation compatibility govern the wall-thickness-dependent tensile response.

## 4. Conclusions

In this study, TA15 alloy walls with thicknesses ranging from 0.5 mm to 30 mm were fabricated by LPBF under identical processing conditions. The wall-thickness-dependent microstructure, thermal history, tensile properties, and fracture behavior were investigated by OM, SEM, EBSD analysis, tensile testing, fractography, and thermal simulation. The main conclusions are as follows:

(1) The LPBF-fabricated TA15 walls are mainly composed of acicular α′ martensite over the investigated wall-thickness range. With increasing wall thickness, the α′ laths coarsen markedly, and the average lath width increases from approximately 0.28 μm in the 0.5-T specimen to 1.55 μm in the 30-T specimen. Meanwhile, the lath morphology evolves from a fine interwoven network to a coarser colony-like structure with enlarged similarly oriented regions.
(2) Wall thickness changes the post-solidification cyclic reheating behavior during LPBF. As wall thickness increases, both the simulated reheating temperature and the cumulative elevated-temperature residence time increase because of stronger heat accumulation. The simulated reheating temperature rises from approximately 150–250 °C for the 0.5-T specimen to 400–550 °C for the 30-T specimen, while the effective thermal exposure increases from the millisecond–second scale to several tens of seconds. The wall-thickness-dependent tensile-property evolution is strongly correlated with cyclic-reheating-induced α′/α lath coarsening, lath arrangement evolution, and deformation compatibility.
(3) The tensile response varies with wall thickness. The yield strength reaches the highest value of 972.3 ± 5.29 MPa in the 1-T specimen, while the highest average ultimate tensile strength of 1113.3 ± 9.0 MPa is obtained in the 2-T specimen. The elongation increases from 9.5 ± 0.6% in the 0.5-T specimen to 17.8 ± 1.7% in the 30-T specimen. Fractography shows that all specimens fail mainly by ductile dimple fracture. With increasing wall thickness, the dimples become larger and deeper, indicating improved plastic-deformation accommodation.
(4) The wall-thickness-dependent strength–ductility balance is governed by the competition between α′ lath-boundary strengthening and deformation accommodation. The 1-T specimen exhibits the highest yield strength because its fine and continuous lath network provides effective barriers to dislocation motion. In thicker walls, α′ lath coarsening and colony-like lath development reduce the boundary density but improve deformation compatibility, leading to higher elongation. Taken together, the wall-thickness effect in LPBF-fabricated TA15 alloy is closely associated with post-solidification cyclic thermal exposure, α′ lath coarsening, and lath-network evolution.

## Figures and Tables

**Table 1:** Nominal chemical composition of the TA15 titanium alloy powder [31].

Element	Al	V	Zr	Mo	Si	C	Fe	O	N	H	Ti
Content (wt.%)	6.09	1.25	2.16	0.81	0.023	<0.20	0.024	0.073	0.0092	<0.0017	Bal.

**Table 2:** Temperature-dependent thermophysical properties of TA15 titanium alloy [31].

Temperature (°C)	Density (kg/m^3^)	Thermal Conductivity (W/(m·°C))	Heat Capacity (J/(kg·°C))
20	4450	8	510
100	4450	8.8	545
200	4450	10.2	587
300	4450	10.9	628
400	4450	12.2	670
500	4450	13.8	712
600	4450	15.1	755
700	4450	16.8	838
800	4450	18	880
900	4450	19.7	922

**Table 3:** Temperature-dependent thermophysical properties of Ti6Al4V titanium alloy [31].

Temperature (°C)	Density (kg/m^3^)	Thermal Conductivity (W/(m·°C))	Heat Capacity (J/(kg·°C))
20	4420	7	546
205	4395	8.75	584
500	4350	12.6	651
995	4282	22.7	753
1100	4267	19.3	641
1200	4252	21	660
1600	4198	25.8	732
1650	3886	83.5	831
2000	3818	83.5	831

**Table 4:** EBSD indexing rate, indexed phase fractions and unindexed fractions of LPBF-fabricated TA15 alloy walls with different thicknesses.

Phase Content (%)	0.5-T	1-T	2-T	5-T	10-T	30-T
Indexing rate	99.4	98.2	99.6	99.5	96.8	99.9
α phase	99	97.8	99.1	99.1	96.5	99.7
β phase	0.4	0.4	0.5	0.4	0.3	0.2
Unindexed fraction	0.6	1.8	0.4	0.5	3.2	0.1

**Table 5:** Average tensile properties and standard deviations of LPBF-fabricated TA15 alloy walls with different thicknesses.

Specimen	0.5-T	1-T	2-T	5-T	10-T	30-T
Measured gauge width (mm)	1.99, 1.99, 2.00	1.98, 1.98, 1.98	1.98, 1.98, 1.98	2.00, 2.01, 1.95	1.97, 1.98, 1.98	2.01, 2.01, 2.00
Measured gauge thickness (mm)	0.90, 0.93, 0.93	0.86, 0.89, 0.83	1.03, 1.03, 1.03	0.99, 0.97, 0.98	0.94, 0.95, 0.99	1.05, 1.04, 1.05
Gauge cross-sectional area (mm^2^)	1.79, 1.85, 1.86	1.70, 1.76, 1.64	2.04, 2.04, 2.04	1.98, 1.95, 1.91	1.85, 1.88, 1.96	2.11, 2.09, 2.10
Yield strength	929 ± 27.2	972.3 ± 5.29	969.3 ± 18.4	943.3 ± 12.6	928.3 ± 9.0	906 ± 11.3
Ultimate strength	1079 ± 25.6	1090.3 ± 5.5	1113.3 ± 9.0	1090 ± 19.0	1083.3 ± 4.7	1068.3 ± 8.1
Elongation	9.5 ± 0.6	9.8 ± 1.4	14.3 ± 0.6	13.7 ± 1.5	13.1 ± 0.2	17.8 ± 1.7

## Data Availability

The original contributions presented in this study are included in the article. Further inquiries can be directed to the corresponding author.
